# Ramsay Hunt syndrome and mandibular alveolar bone necrosis following herpes zoster: A case report and literature review

**DOI:** 10.3389/fneur.2022.1073607

**Published:** 2022-12-15

**Authors:** Maojia Yin, Panchuan Huang, Sen Yang, Wuchao Wang

**Affiliations:** ^1^Department of Pain Medicine, Daping Hospital, Army Medical University, Chongqing, China; ^2^Department of Stomatology Medicine, Daping Hospital, Army Medical University, Chongqing, China

**Keywords:** herpes zoster, Ramsay Hunt syndrome, pain, teeth exfoliation, jaw osteonecrosis, trigeminal nerve

## Abstract

**Background:**

Reactivation of latent varicella-zoster virus (VZV) can induce herpes zoster (HZ). Ramsay Hunt syndrome (RHS) occurs through the reactivation and proliferation of VZV in the geniculate ganglion, which can lead to vesicular rash in the ear or oral mucosa, accompanied by neurological disorders.

**Materials and methods:**

A 50-year-old man sought a remedy for pain in the right ear and face. Within 1 week, all his lower right teeth fell out, and in the following 3 months, his lower right mandibular alveolar bone gradually became necrotic. In the past 20 days, he experienced blister rash, hearing and taste loss, and slight facial paralysis.

**Results:**

After ruling out tumors and other infectious diseases, he was diagnosed with trigeminal HZ and RHS.

**Conclusion:**

Ramsay Hunt syndrome with tooth loss and alveolar osteonecrosis is rare. It requires long-term treatment of pain, and prevention and treatment of tooth loss and alveolar bone necrosis are difficult and warrant further study.

## Introduction

Primary infection with varicella-zoster virus causes chickenpox, followed by viremia with a diffuse rash and latency in the cranial nerve, dorsal root, and autonomic ganglia. Reactivation of latent VZV can induce HZ, which spreads from the cranial nerve or dorsal root ganglia to the dermatome along the sensory nerves. In the USA, >1 million people annually have HZ, with an annual rate of three or four cases per 1,000 persons (1, 2). In China, the prevalence of HZ is 7.7% in hospital patients aged ≥40 years (3). The incidence rate of HZ in the unvaccinated elderly (50 years old) is ~10/1000 person-years (1).

Ramsay Hunt syndrome occurs by the reactivation and multiplication of VZV at the geniculate ganglion, which leads to a vesicular rash on the ear or in the oral mucosa, combined with neurological disturbances (4). Here, we report a rare case of trigeminal HZ combined with RHS, mandibular alveolar bone necrosis, and tooth loss on the same side.

## Case report

**Figure 1:**
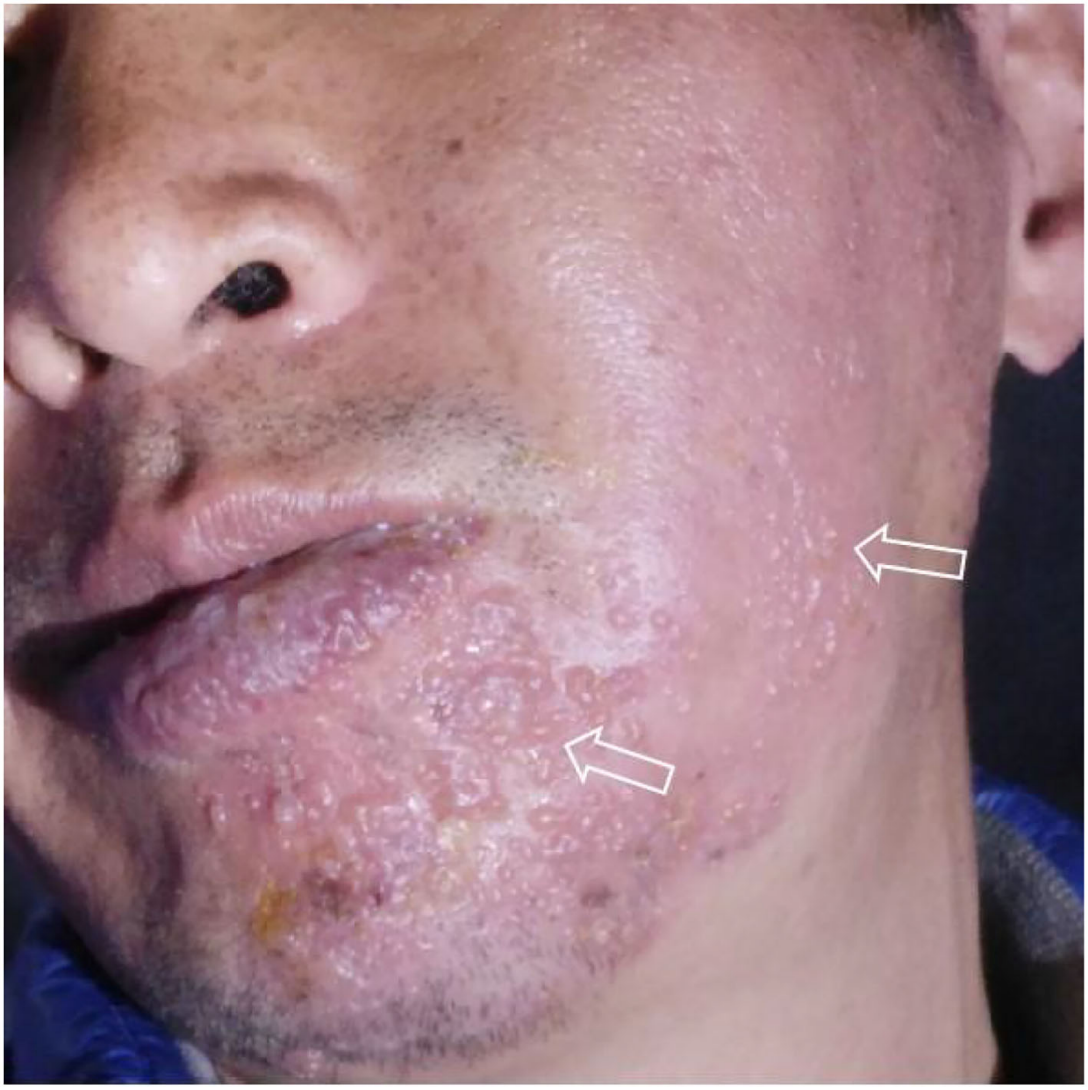
Typical unilateral aggregated vesicular rash at the beginning of the disease (2021.4.13), which originated from the patient's self-photography (mirror image).

A 50-year-old man presented to our pain department with a 7-day history of severe acupuncture-like pain in the right prefrontal, temporal, cheek, and mandibular regions and right ear, especially in the mandible, and four right lower teeth fell out within 1 week. In the past 20 days, he experienced blister rash (Figure 1), hearing and taste loss, and slight facial paralysis (Table 1). He had no symptoms of vertigo, tinnitus, nausea, or vomiting. Based on the initial evaluation, the pain intensity was rated as 8 on the visual analogue scale (VAS).

**Table 1:** Medical history before admission.

**Time^a^**	**Clinical manifestation**	**Medical institution**	**Diagnosis**	**Treatment**	**Prognosis**
20 days before (2021.04.08)	Pain: persistent mild pain in the right lower tooth; VAS:2. Rash: right oral mucosa and gingival blisters and erosion.	Dental clinic	Mouth ulcer	Metronidazole gargle/2 days	No improvement
15 days before (2021.04.13)	Pain: persistent mild pain in right lower tooth; VAS:2. Rash: severe vesicular eruption started from the lower right lip and chin and extended to the face, forehead and ear. Function: right hearing loss, taste loss and facial paralysis (House–Brackman grade III)^b^.	Hospital	HZ	Intravenous infusion of methylprednisolone, ceftriaxone and acyclovir/5 days.	Pain: no pain; VAS:0. Rash: gradually scabbed and recovered Function: normal hearing and taste, facial paralysis improved (House–Brackman grade II)^c^
7 days before (2021.04.21)	Pain: severe pain in the right rash area, especially in the mandible; VAS:8. Function: four right lower teeth fell out within 1 week.	None	/	Diclofenac sodium sustained release tablets, 0.1g per day/7 days.	No improvement

^a^Days before visiting our department.

^b^When talking with family members, they need to speak louder; taste was weaker than before; the patient's mouth was tilted to the left and food accumulated on the right side of the mouth when eating (House–Brackmann grade III).

^c^Right nasolabial groove was shallower than the opposite groove (House–Brackmann grade II).

The patient had been smoking for >30 years and smokes at least 20 cigarettes a day. The patient was healthy in the past and had no complications. He did not take any drugs other than those mentioned in Table 1. There was no specific abnormality in his family genetic history.

Intraoral examination revealed severe loosening of the lower right second premolar, and first, second and third molars. The lower right central incisor, lateral incisor, canine, and first premolar had exfoliated, leaving non-healing sockets. The oral hygiene was poor with generalized gingivitis. Extraoral examination demonstrated marked patchy scars of the right facial skin including the forehead, right ear and cheek, angle of the mouth, congestion and swelling of the right external auditory canal, attachment of purulent secretions, and congestion and swelling of the tympanic membrane. His right nasolabial groove is shallower than the opposite, and facial paralysis was classified as House–Brackmann grade II.

**Figure 2:**
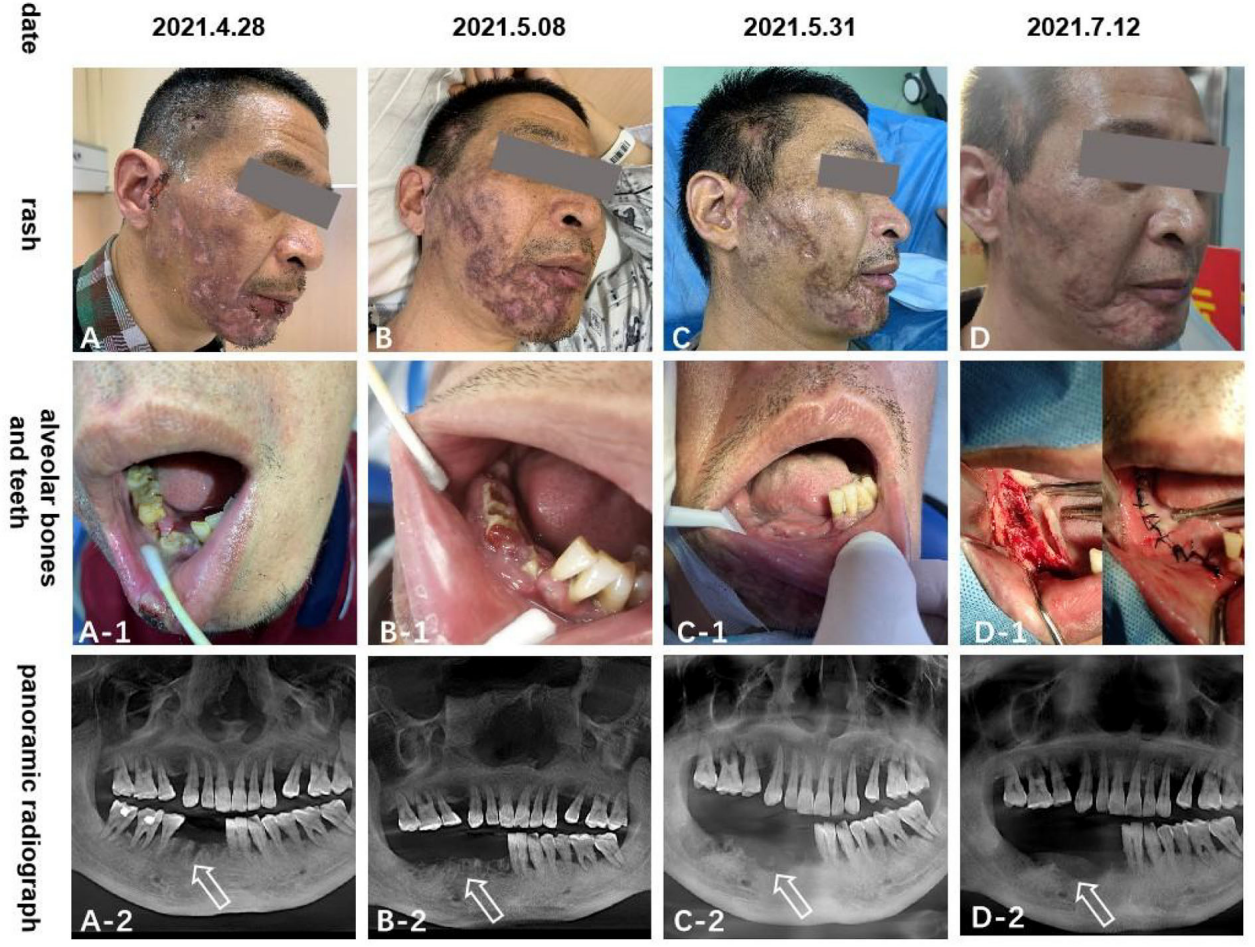
The **(A–D)** pictures show the rash scar gradually becomes shallow; **(A-1–D-1**) and **(A-2–D-2)** pictures show that the right mandibular teeth gradually fall off and the alveolar bone gradually necrosis.

**Figure 3:**
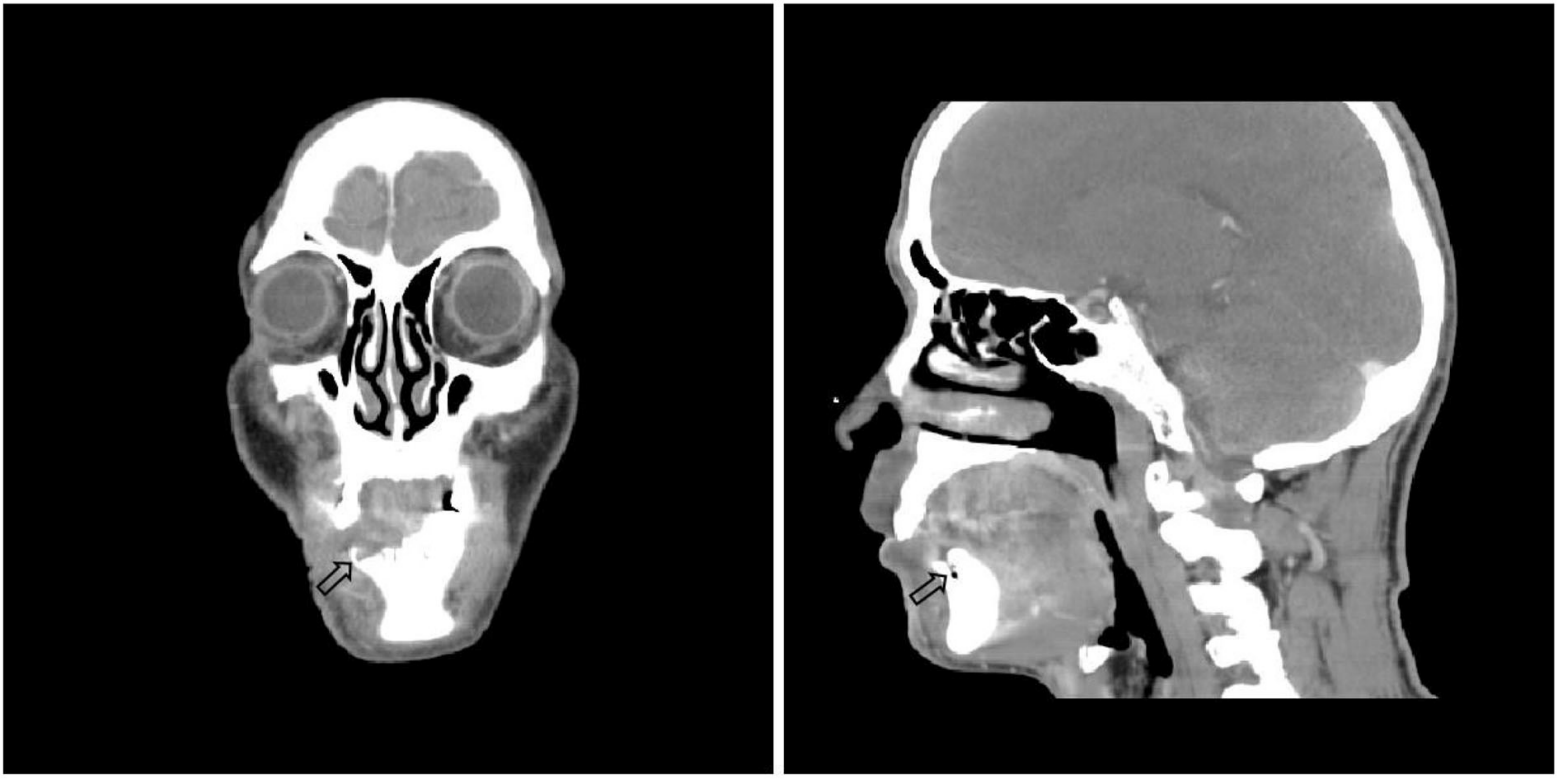
Cranial neck enhanced CT(2021-4-30) showed the right side of the lower alveolar bone is partially absorbed, and there is no corresponding tooth. No tumor or other suspicious lesions.

There were no obvious abnormalities in renal function and coagulation. Routine blood tests showed a neutrophil percentage of 77.1%, total neutrophil count of 8.06 × 10^9^/L, erythrocyte sedimentation rate of 35.0 mm/h, and inflammatory marker IL-6 of 10.31 pg/ml. Syphilis, HIV, hepatitis B virus, and hepatitis C virus tests were all negative. Anaerobic and aerobic bacterial cultures of skin exudates from ulcerative infection were negative. The following tumor markers were negative: alpha-fetoprotein, carcinoembryonic antigen, carbohydrate antigen (CA) 153, total and free prostate-specific antigen, CA199, CA24-2, and neuron-specific enolase. Autoimmune anticyclic citrullinated peptide, antikeratin, and antinuclear antibodies were negative. Panoramic radiography showed that the lower right central incisor, lateral incisor, canine, and first premolar had exfoliated (Figure 2A-2). Cranial neck enhanced CT showing osteonecrosis in the right anterior mandible (Figure 3). Electronic fiber otoscopy showed a blistering rash in the external auditory canal.

The rash in our patient was characterized by unilateral aggregated vesicles distributed along the nerves (Figures 1, 2), and the corresponding nerves had different degrees of dysfunction, so we made a diagnosis of HZ. Based on the patient's history of hearing and taste loss and facial paralysis in the early stage, tooth loss and mandibular pain in the late stage, and the distribution of the rash in the right ear canal and on the head and face, we considered that the case involved cranial nerves V, VII, and VIII; therefore, we diagnosed it as RHS.

In terms of treatment, we followed the recommendations of the relevant guidelines (5, 6). When gabapentin measurement reached 0.3 g three times a day for 4 days and the pain could not be relieved, we switched to pregabalin 150 mg two times daily, paracetamol tramadol tablets 362.5 g three times daily, and eperisone hydrochloride 75 mg three times daily, orally for 7 days. As a result of poor relief of jaw pain, we considered that the trigeminal nerve was involved, so we added carbamazepine 0.1 g two times daily orally for 7 days to relieve the pain. Exposed necrotic bone was trimmed periodically; cefixime and metronidazole oral prophylaxis were administered; physiological saline was used for ear canal irrigation and levofloxacin ear drops, two drops three times daily for 10 days. At the time of discharge, the pain score decreased from 8 to 4 on the VAS, and the skin rash healed, leaving a residual scar. The right lower teeth completely fell out (Figure 2).

One month after discharge, the patient underwent a mental foramen nerve block in our outpatient department due to the increased mandibular pain, which reduced the VAS score from 4–6 to 3–4, and after medication, the VAS score gradually decreased to around 2 after 3 months. Since then, we began to gradually reduce the oral drug dose until the pain disappeared (about 6 months later). The right mandible gradually became necrotic, and mandibular debridement was performed regularly in the Department of Stomatology in our hospital until it almost disappeared after 3 months (Figure 2). At the last follow-up visit (6 months later), the pain had disappeared, and the lower right alveolar bone was almost completely necrotic. The facial scar healed and the left teeth were used for chewing.

## Discussion

Herpes zoster can occur in anyone who has previously been infected with VZV, through the reactivation of the virus (4). Viral reactivation often occurs in immunocompromised individuals, such as those with physical and psychological trauma, tumor, hematological disease, diabetes, HIV infection, immunosuppressive therapy, malnutrition, aging, emotional stress, smoking, depression, organ transplantation, and some drugs, including but not limited to corticosteroids and immunosuppressive agents (4, 7). Our patient was older, had a long history of smoking, and suffered from depression.

Ramsay Hunt syndrome is caused by the reactivation of VZV in the geniculate ganglion, with an incidence of 1% (8). Affected patients can have a blistering rash in the ear (zoster auricularis) or oral mucosa, accompanied by acute peripheral facial paralysis. Other manifestations include pain (face, ears, temporomandibular joint, or teeth); hearing, vision, and taste disorders; tinnitus; vertigo; nausea; ptosis of upper eyelid; increased nasal secretions; fever and discomfort; and abnormal tactile sensation, which are often associated with the involvement of the cranial nerves, such as V, IX, XI, and XII (9).

Recalling the patient's medical history, we speculate that the dentist at the clinic misdiagnosed the oral mucosal herpes as an oral ulcer in the earliest stages. Combined with the clinical manifestations of the patient, the most appropriate description is that HZ was associated with cranial nerve V involvement (mandibular pain and right lower tooth loss), cranial nerve VII involvement (facial paralysis and taste loss), and cranial nerve VIII involvement (hearing loss). The symptoms of cranial nerves VII and VIII involvement improved quickly in the early stage of the disease, which may have been because hormone therapy was used at the initial stage of inflammation.

The pathogenesis of osteonecrosis during HZ was still debated and seems to be multifactorial. Meer et al. (10) suggested that this may be due to the compression of the alveolar artery in the narrow bone canal after edema caused by nerve infection, resulting in arterial ischemia and necrosis of the supply area. Mendieta et al. (11) suggested that the virus spreads directly from adjacent cranial nerves and invades blood vessels, causing segmental granulomatous vasculitis, which seriously affects the growth of the supply area. Judging from the large area of acute skin lesions, our patient's nerves and blood vessels should have been attacked by a large number of viruses, which may have produced the same features as above, leading to tooth loss. Periodontal diseases, such as periodontitis or pulpitis, are common causes of jaw osteonecrosis (12). Badjate et al. (13) explained that tooth exfoliation is an early sign of post-herpetic osteonecrosis. The loss of dental proprioception caused by damage to periosteal blood flow leads to periodontal membrane necrosis before alveolar osteonecrosis. Similarly, our patient's hygiene was poor, and he had generalized gingivitis, which may also be one of the reasons for tooth loss and alveolar bone necrosis. Also, the vasoconstriction caused by sympathetic excitation should not be ignored (14). The sympathetic nervous system has a vasoconstrictive effect on the peripheral vascular bed, which is regulated by the local vascular nerve signaling mechanism. When the blood vessels or sympathetic nerves are invaded by the virus, they are stimulated to produce a vasoconstrictive response, which can lead to the obstruction of blood supply to local tissues. It has also been suggested that some neural mechanisms may be involved. Some studies have shown genetic, neuroanatomical, and physiological evidence that leptin regulates bone mass by regulating sympathetic nerve tension (15).

Early application of antiviral drugs and antibiotics combined with aggressive debridement of necrotic bone is considered to improve wound healing to the greatest extent (16). Some scholars have recommended that low-dose hormones combined with antiviral treatment in the early stage of the disease can improve the symptoms associated with viral invasion of the nerves (17). However, mandibular necrosis occurred in a 30-year-old female patient with lupus erythematosus who took prednisone (5 and 7.5 mg, alternately) for 9 years and a 50-year-old male patient with psoriatic arthritis who took prednisone (7.5 mg/day) for 2 years (18, 19). We believe that the key to mandibular necrosis caused by hormone application is long-term use, and we do not use hormone treatment anymore. In recent studies, complementary treatment, including platelet concentrates in solid and liquid form, has been developed to prevent jaw osteonecrosis and improve healing after surgical treatment of bone lesions (19). During treatment, we should pay attention to screening for occult malignancies, immunodeficiency, and other systemic conditions, so as to make the treatment more comprehensive (20). To enhance immunoprotection, Cunningham et al. have recommended the administration of two doses of recombinant virus and adjuvant varicella-zoster vaccine, 2 months apart, beginning at age 60 years (21). Thus, vaccination is an important way to avoid the occurrence of herpes zoster.

## Conclusion

Herpes zoster is characterized by pain and neurological impairment in addition to rashes. We need to pay close attention to its medical history when treating such patients to better identify the involved nerves. Our patient had a rare case of RHS with tooth loss and jaw necrosis. There are many references in the literature to the treatment of pain and rash. However, the treatment of tooth loss and jaw necrosis is limited to symptomatic treatment. It is hoped that more cases can be seen to investigate the pathogenetic mechanism and to find timely preventive methods to improve the prognosis of patients.

## Data availability statement

The original contributions presented in the study are included in the article/supplementary material, further inquiries can be directed to the corresponding author/s.

## Ethics statement

The studies involving human participants were reviewed and approved by the Ethics Committee of PLA Army Characteristic Medical Center; Army Medical University. The patients/participants provided their written informed consent to participate in this study. Written informed consent was obtained from the individual(s) for the publication of any potentially identifiable images or data included in this article.

## Author contributions

All authors listed have made a substantial, direct, and intellectual contribution to the work and approved it for publication.
